# Neoinnervation and neovascularization of acellular pericardial-derived scaffolds in myocardial infarcts

**DOI:** 10.1186/s13287-015-0101-6

**Published:** 2015-05-27

**Authors:** Carolina Gálvez-Montón, M. Teresa Fernandez-Figueras, Mercè Martí, Carolina Soler-Botija, Santiago Roura, Isaac Perea-Gil, Cristina Prat-Vidal, Aida Llucià-Valldeperas, Ángel Raya, Antoni Bayes-Genis

**Affiliations:** ICREC Research Program, Fundació Institut d’Investigació en Ciències de la Salut Germans Trias i Pujol, Camí de les Escoles s/n, Badalona, Barcelona 08916 Spain; Pathology Department, Hospital Universitari Germans Trias i Pujol Ctra. Canyet, s/n,, Badalona, Barcelona 08916 Spain; Center of Regenerative Medicine in Barcelona, Dr. Aiguader, 88, Barcelona, 08003 Spain; Centro de Investigación Biomédica en Red de Bioingeniería, Biomateriales y Nanomedicina (CIBER-BBN), Baldiri Reixac, 10, Barcelona, 08028 Spain; Institute for Bioengineering of Catalonia (IBEC) and Institució Catalana de Recerca i Estudis Avançats (ICREA), Barcelona, Spain; Department of Medicine, Universitat Autònoma de Barcelona (UAB), Ctra. de Canyet, s/n, Barcelona, Spain 08916; Cardiology Service, Hospital Universitari Germans Trias i Pujol, Badalona, Barcelona Spain

## Abstract

Engineered bioimplants for cardiac repair require functional vascularization and innervation for proper integration with the surrounding myocardium. The aim of this work was to study nerve sprouting and neovascularization in an acellular pericardial-derived scaffold used as a myocardial bioimplant. To this end, 17 swine were submitted to a myocardial infarction followed by implantation of a decellularized human pericardial-derived scaffold. After 30 days, animals were sacrificed and hearts were analyzed with hematoxylin/eosin and Masson’s and Gallego’s modified trichrome staining. Immunohistochemistry was carried out to detect nerve fibers within the cardiac bioimplant by using β_III_ tubulin and S100 labeling. Isolectin B4, smooth muscle actin, CD31, von Willebrand factor, cardiac troponin I, and elastin antibodies were used to study scaffold vascularization. Transmission electron microscopy was performed to confirm the presence of vascular and nervous ultrastructures. Left ventricular ejection fraction (LVEF), cardiac output (CO), stroke volume, end-diastolic volume, end-systolic volume, end-diastolic wall mass, and infarct size were assessed by using magnetic resonance imaging (MRI). Newly formed nerve fibers composed of several amyelinated axons as the afferent nerve endings of the heart were identified by immunohistochemistry. Additionally, neovessel formation occurred spontaneously as small and large isolectin B4-positive blood vessels within the scaffold. In summary, this study demonstrates for the first time the neoformation of vessels and nerves in cell-free cardiac scaffolds applied over infarcted tissue. Moreover, MRI analysis showed a significant improvement in LVEF (*P* = 0.03) and CO (*P* = 0.01) and a 43 % decrease in infarct size (*P* = 0.007).

**Electronic supplementary material:**

The online version of this article (doi:10.1186/s13287-015-0101-6) contains supplementary material, which is available to authorized users.

## Findings

### Background

Myocardial infarction (MI) was first described over a century ago, yet it remains a leading cause of death worldwide despite the significant advances achieved in recent years [1]. In this context, cardiac tissue engineering is a new discipline of growing interest for rebuilding and regenerating myocardial necrosis after ischemic events [2]. To ensure the effectiveness of engineered bioimplants, they should be electromechanically coupled with the host myocardium and supported by functional vasculature and innervation to produce viable and stable contractile function [3]. Non-innervated cardiac bioimplants may lead to incomplete integration with the surrounding cardiac tissue, which is innervated by the autonomic nervous system [4]. Currently, the *de novo* innervation of cardiac engineered bioimplants is incompletely characterized and little is known about its occurrence after MI. Accordingly, we studied the presence of nerve sprouting and neovascularization in a cell-free pericardial-derived scaffold implanted over a post-infarct scar in swine after 30 days of follow-up.

## Methods

A detailed description of the experimental process and analysis is provided in Additional file 1 of the supplemental material. This study was approved by the Minimally Invasive Surgery Centre Jesús Usón Animal Experimentation Unit Ethical Committee (#ES 100370001499) and complies with all guidelines concerning the use of animals in research and teaching as defined by the Guide for the Care and Use of Laboratory Animals (National Institutes of Health Publication #80–23, revised 1996). Human pericardial samples were acquired after written informed consent was obtained from all patients undergoing cardiac surgery. The Germans Trias i Pujol University Hospital ethics committee approved this study (PI-14-050), and the protocol conformed to the principles outlined in the Declaration of Helsinki.

Briefly, 17 animals were submitted to an MI. Thirty minutes later, a decellularized pericardial-derived scaffold, rehydrated with RAD16-I, was placed over the ischemic myocardium as previously described [5]. After sacrifice, the hearts were excised and analyzed with hematoxylin/eosin (H/E) and Masson’s and Gallego’s modified trichrome staining. Immunohistochemistry was carried out to detect nerve fibers within the cardiac bioimplant by using β_III_ tubulin and S100 labeling. Isolectin B4, smooth muscle actin (SMA), CD31, von Willebrand factor (vWF), cardiac troponin I, and elastin antibodies were used to study scaffold vascularization. Moreover, transmission electron microscopy (TEM) was performed to confirm the presence of vascular and nervous ultrastructures. Additionally, seven animals were submitted to magnetic resonance imaging (MRI) to measure the effect of the pericardial-derived scaffold on cardiac function. Functional parameters were monitored at baseline, 48 h post-MI, and after 1 month, before sacrifice. Left ventricular ejection fraction (LVEF), cardiac output (CO), stroke volume, end-diastolic volume, end-systolic volume, end-diastolic wall mass, and infarct size measurements were blindly analyzed. Statistical analysis was performed with SPSS statistic software (19.0.1 version; SPSS Inc., Chicago, IL, USA). Differences in MRI analysis were analyzed by using Student’s *t* test for paired samples. Data are presented as the mean ± standard error of the mean, and statistical significance was achieved when *P* <0.05.

## Results and Discussion

**Fig. 1:**
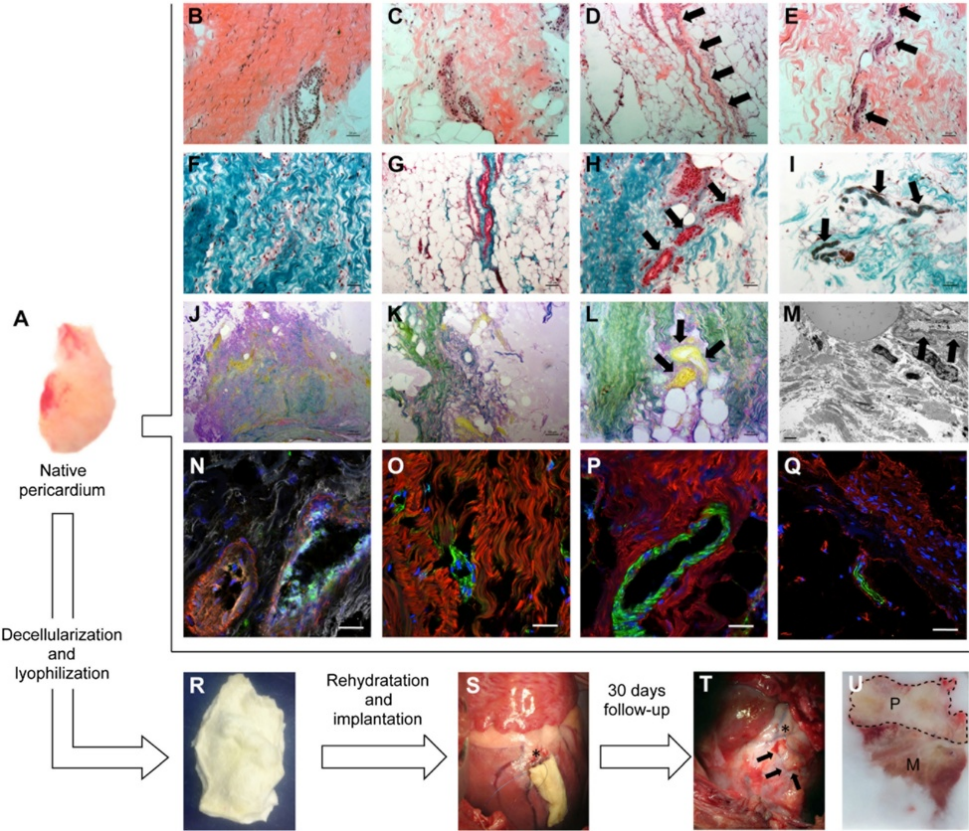
Native human pericardium. **a** Native human pericardium. **b**-**l** The corresponding sections were composed of dense tissue rich in collagen and, elastin, and adipose tissue after hematoxylin/eosin staining (**b** and **c**, respectively), light green Masson’s (**f** and **g**, respectively), and Gallego’s modified trichrome (**j** and **k**, respectively) staining. Vascular (**d**, **h** and **l**) and nerve (**e** and **i**) structures in native human pericardium. **m** Transmission electron microscopy image of native pericardium showing the presence of microvasculature (*arrows*). Blood vessels in human pericardium labeled with isolectin B4 (*green*), smooth muscle actin (*red*), and elastin (*white*) antibodies (**n**), von Willebrand factor (*green*) and collagen I (*red*) (**o**), and CD31 (*green*) and collagen I (*red*) (**p**). **q** Nerve fibers in the native pericardium after β_III_ tubulin (*green*) and elastin (*red*) labeling. Nuclei are counterstained with 4′,6-diamidino-2-phenylindole (*DAPI*) (blue). **r** Decellularized and lyophilized human pericardium and (**s**) its implantation after coronary ligature (*asterisk*). **t** Photograph of a decellularized, cell-free pericardium 30 days after implantation. **u** Snap-frozen sample including the myocardium (*M*) and the pericardial scaffold (*P*) adhered to the cardiac muscle. Scale bars = 100 μm (D, G, J and K), 50 μm (b, c, e, f, h, i, l, o, p, q, and r), and 2 μm (**m**)

First, we examined the composition of native human pericardial tissue (Fig. 1a), which was snap-frozen, and 10-μm sections were made. To describe the histological structure of this tissue, it was subjected to H/E and Masson’s and Gallego’s modified trichrome staining. The morphologic structure of the pericardium had two main domains: (1) an extracellular matrix (ECM) rich in collagen and elastin fibers (Figs. 1b, f, and j) and (2) a cellular domain composed mainly of adipocytes (Figs. 1c, g, and k). Native pericardium includes blood vessels (Figs. 1d, h, and l) and nerve fibers (Figs. 1e and i), as confirmed by the presence of isolectin B4-positive vessels, with and without SMA (Figs. 1n and o), and nerve fibers labeled with β_III_ tubulin (Figs. 1p and q). Vascular ultrastructures were also observed in TEM images (Fig. 1m).

Next, the pericardium was subjected to a complete decellularization, lyophilization, and sterilization process (Fig. 1r), resulting in a newly engineered pericardial-derived scaffold ready to apply over the MI (Fig. 1s). Figure S1 of Additional file 2 shows pericardial-derived scaffolds free of nuclei and cellular debris preserving its ECM. Furthermore, a previous work showed that, after detergent treatment, scaffold DNA content was significantly lower than native pericardium; reduction was approximately 70 % [5].

Twenty-one animals underwent to a transmural MI by final artery ligation. During follow-up, one swine died of ventricular fibrillation and three were excluded from the study because of local scaffold infection. The remaining 17 animals were sacrificed after 28.45±4.25 days of follow-up. Before sacrifice, the heart was exposed and the pericardial-derived scaffold covering the myocardium was examined in all cases (Fig. 1t). Then this area was excised, paraffin-embedded, or snap-frozen (Fig. 1u).

**Fig. 2:**
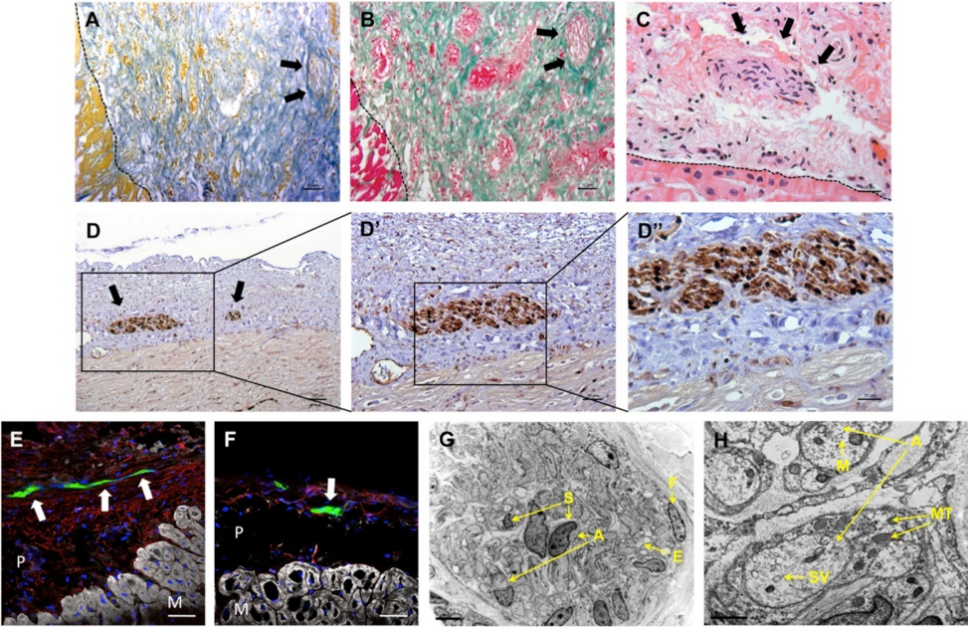
Neoinnervation of pericardial-derived scaffolds after myocardial infarction. Histological sections of the pericardial-derived scaffold showing nerve fibers (*arrows*) after Gallego’s modified trichrome (**a**), light green Masson’s trichrome (**b**), and hematoxylin/eosin (**c**) staining. **d**-**d′′** Immunohistological analysis exhibiting positive S100 nerve structures in the scaffold and corresponding zoomed images. **e** and **f** Immunofluorescence against β_III_ tubulin (*green*), cardiac troponin I (*white*), and elastin (*red*) labeled nerve fibers (*arrows*), myocardium (*M*) and pericardial-derived bioimplant (*P*), respectively. Nuclei are counterstained with 4′,6-diamidino-2-phenylindole (*DAPI*) (blue) (**g** and **h**). Transmission electron microscopy images of the scaffold showing nerve structures composed of Schwann cells (*S*), axons (*A*), and fibroblasts of the perineurium (*F*), endoneurium (*E*), transport vesicles (*TV*), microtubules (*M*), mitochondria (*MT*), and synaptic vesicles (*SV*). Scale bars = 100 μm (d), 50 μm (a, b, d′, e, and f), 20 μm (c and d′′), 5 μm (g), and 2 μm (h)

**Fig. 3:**
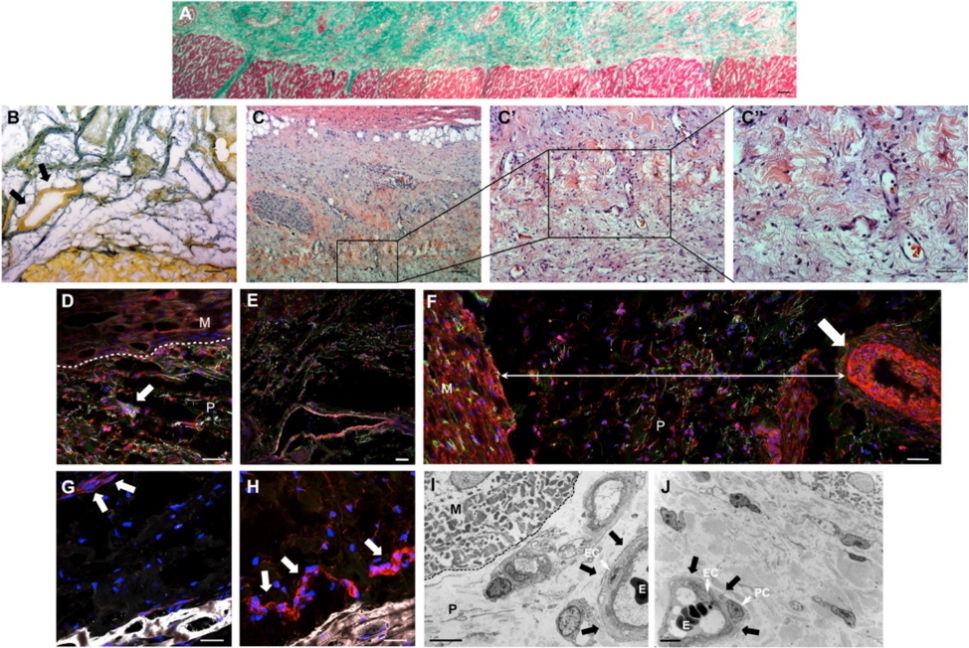
Neovascularization of pericardial-derived scaffolds after myocardial infarction. **a** Masson’s trichrome staining composition screening demonstrated the correct adhesion of the cardiac bioimplant with the adjacent myocardium. **b** and **c**-**c′′** Gallego’s modified trichrome and hematoxylin/eosin staining exhibiting vessels in the cardiac matrix and zoomed images, respectively. Immunofluorescence analysis in the pericardial-derived scaffold after its implantation against isolectin B4 (*green*), smooth muscle actin (*red*), and elastin (*white*) indicates the presence of microvasculature (**d**), venules (**e**), and arterioles (**f**). Red positive immunostaining against CD31 (**g**) and von Willebrand factor (**h**) antibodies in pericardial-derived scaffold (*arrows*). Nuclei are counterstained with 4′,6-diamidino-2-phenylindole (*DAPI*) (blue) (**i** and **j**). Transmission electron microscopy images of the pericardial-derived scaffold show vascular structures (*arrows*) where endothelial cells (*EC*), pericytes (*PC*), and erythrocytes (*E*) are present. *M* myocardium, *P* pericardial-derived scaffold. Scale bars = 100 μm (a-c), 50 μm (c′ and d-h), 20 μm (c′′), and 5 μm (i and j)

H/E and Masson’s and Gallego’s modified trichrome staining provided a histological description of the pericardial-derived scaffold. The presence of *de novo* nerve fibers (Figs. 2a-c) and blood vessels (Figs. 3a-c) within the scaffold was confirmed. Moreover, Fig. 3a illustrates perfect adhesion and integration of the scaffold with the adjacent myocardium. Immunohistochemistry showed newly formed nerve fibers positive for S100 protein (Figs. 2d-d′′) and β_III_ tubulin (Figs. 2e and f). Additionally, TEM images helped to discern nerve structures composed of several amyelinated axons (Fig. 2g), numerous transport vesicles, microtubules, and mitochondria (Fig. 2h).

The examination of vascular structures under immunohistochemistry demonstrated that *de novo* small (Fig. 3d) and large (Fig. 3e) isolectin B4-positive blood vessels were also found within the scaffold. Indeed, specific SMA identified arterioles with a thick SMA-positive layer (Fig. 3f) and venules with a thin muscular layer (Fig. 3e). Of note, as shown in Fig. 3f, neovessels were dispersed all over the thickness of the scaffold, often located away from the adjacent myocardium. These new-formed vessels were also positive for CD31 (Fig. 3g) and vWF (Fig. 3h) antibodies. TEM better characterized the vascular structures within the scaffold and the presence of erythrocytes within neovessel lumen, confirming functional conduits with blood flow (Figs. 3i and j). Capillaries were identified by endothelial cells surrounded by pericytes.

**Fig. 4:**
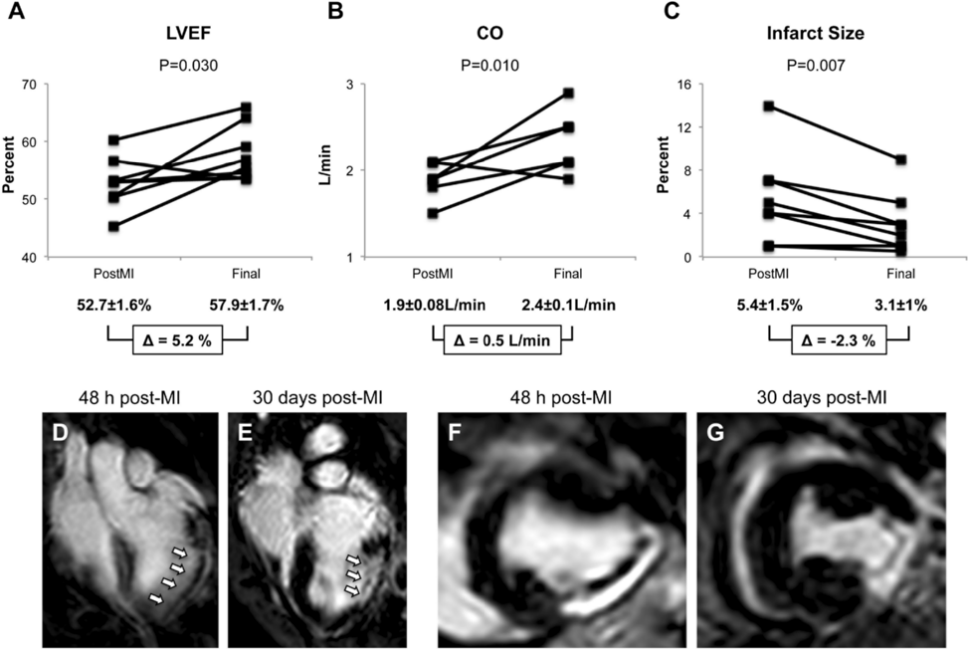
Cardiac function analysis by magnetic resonance imaging. Left ventricular ejection fraction (*LVEF*) (**a**), cardiac output (*CO*) (**b**), and infarct size (**c**) at 48 h and 30 days after myocardial infarction (*MI*) and decellularized pericardial-derived scaffold implantation. Data for individual pigs (*dots*) are shown. *P* = 0.030 (a), *P* = 0.010 (b), and *P* = 0.007 (c). Representative turbo-spin-echo (**d** and **e**) and T1 short-axis delayed enhancement (**f** and **g**) images from one pig 48 h and 30 days after MI and decellularized pericardial-derived scaffold grafting. *Arrows* indicate MI in the left ventricular wall, and T1 images show healthy myocardium (*black*) and the MI with gadolinium retention (*brilliant white*)

Cardiac function analysis after MRI showed significant differences between 48 h post-MI and final data in LVEF (52.7±1.6 versus 57.9±1.7; *P* = 0.03) and CO (1.9±0.1 versus 2.4±0.1; *P* = 0.01) (Figs. 4a and b, respectively). Additionally, delayed enhancement images showed a significant reduction in infarct size (5.4±1.5 versus 3.1±1; *P* = 0.007) (Figs. 4c-g).

This study analyzed for the first time neoinnervation and neovascularization of a cell-free engineered scaffold implanted in swine after an induced MI and its effect in cardiac function. This newly engineered pericardial-derived scaffold was recently shown to integrate well as a cardiac bioimplant after an acute MI in swine [5]. In the present work, we decided to use cell-free scaffolds to unambiguously analyze the contribution of host-derived cells rather than that of cells delivered through the scaffold.

In cardiac tissue engineering, synthetic (e.g., polylactic acid and polyglycolic acid) and biological (e.g., collagen and fibrin) scaffolds have been extensively used [2]. None of these has proven optimal as far as replicating the local tissue-specific architecture. More recently, ECM-derived scaffolds obtained from decellularized natural tissues emerged as promising alternatives since they preserve the natural tunnels necessary for vessels and nerves and the porosity to nest specialized cells. Decellularized native tissues keep their ECM mechanical integrity and bioactive molecules favoring cell-ECM adhesion and cellular contact [6]. To date, decellularized tissues are known to contribute to cell migration, proliferation, and differentiation [7]. It was unclear whether vascularization to ensure oxygen and nutrient diffusion and innervation to support contractile properties and electrical coupling with the target tissues were supported by ECM-derived scaffolds [8].

Prior cardiac tissue engineering studies have already demonstrated scaffold revascularization; in most cases, this was achieved by adding angiogenic factors or embedding progenitor cells within the scaffold with paracrine neovascular effects. Here, we show vascular neoformation in a decellularized scaffold covering ischemic myocardium in the context of acute MI. Neovascularization was demonstrated by isolectin B4-, CD31-, and vWF-positive labeling, and SMA media thickness enabled characterization of arterial or venous traits. Vascular connections between the pericardial-derived scaffold and the underlying myocardium were not identified, but the neovessels were found to contain erythrocytes within their lumen, indicating functional connections with the host vascular system. Finally, the presence of pericytes surrounding the endothelial cells indicates dynamic regulation of this vascular system [9]. Elucidating the exact mechanism(s) by which our scaffolds become vascularized will require further investigation. However, it is likely caused by the local myocardial hypoxic milieu that promotes the expression of vascular endothelial and platelet-derived growth factors, stromal cell-derived factor 1α, and macrophage chemotactic protein, mobilizing host-circulating cells to vascularize the damaged tissue [10]. Moreover, injectable self-assembling peptide nanofibers, such as those formed by RAD16 (used in this study), generate a favorable microenvironment that recruits and promotes survival and self-organization of endothelial cells [11].

Nerve sprouting assessment in cardiac tissue engineering is novel, although pilot studies using adenoviral overexpression of glial-derived neurotrophic factor in the graft tissue [4] or S100A1 gene transfer to strengthen the engineered cardiac grafts have already been reported [12]. S100 proteins are calcium-binding proteins that regulate multiple cellular and molecular functions, including contraction, proliferation, differentiation, and apoptosis in physiological and pathophysiological settings [13–15]. S100-positive nervous fibers in the present study support the identification of differentiated neural cells, such as Schwann cells, in the pericardial-derived scaffolds. Schwann cells are known to stimulate nerve regeneration in both the central and peripheral nervous systems [16]. By contrast, β_III_ tubulin expression is an early marker of neuronal commitment identified in primitive neuroepithelium. Previous studies have shown that hypoxia induces β_III_ tubulin expression in different clinical settings such as glioblastoma, glioma cells, lung and ovarian cancer, and umbilical cord blood-derived mesenchymal stem cells from hypoxic infants [17–19]. In the present work, we also identified MI as a hypoxic model that induces β_III_ tubulin expression and gives rise to neurite outgrowth in the pericardial-derived scaffold. Additionally, TEM images suggest that these newly formed neural cells are unmyelinated as the afferent nerve endings of the heart [20].

Furthermore, after MRI cardiac function analysis, the cell-free engineered scaffold after MI is responsible for important significant improvements in LVEF and CO and a 43 % reduction in infarct size. We may speculate that since the neoinnervated and neovascularizated scaffold is well adapted to the damaged myocardium, this new-engineered bioimplant could be driven to a maturation and functional development, improving the loss of cardiac function after MI [3].

## Conclusions

This study demonstrates neoformation of vessels and nerves in a cell-free cardiac scaffold made of decellularized pericardium after MI in swine. These data suggest that vascularization and innervation processes are supported by the matrix structure, are hypoxia-dependent, and require mobilization of host cells. Thus, the search for an optimal scaffold that preserves the natural tunnels needed for vascular vessels and nerves and with the porosity to nest cells may be crucial to ensure a functional and successful engineered bioimplant. Further studies are needed to explore the mechanisms underlying neovascularization and neoinnervation of an acellular scaffold and to assess the clinical benefits of such phenomena.

### Limitations of the study

Landrace × Large White prepuberal pigs were used and thus results may differ from those of an adult model. For this purpose, it would be interesting to perform further studies at later time points in order to analyze the durability of the biological effects exerted by the pericardial-derived scaffold after MI.

## Additional files

Additional file 1:
**Supplemental Material.**
Additional file 2: Fig. S1. Decellularized human pericardium. Histological sections of cell-free human pericardium after hematoxilin/eosin (**a**), light green Masson’s (**b**), and Gallego’s modified (**c**) trichrome staining. Immunohistofluorescence against β_III_ tubulin (*green*), elastin (*red*), and isolectin B4 (*white*) of a decellularized pericardium showing the absence of nerves, vessels, and cell nuclei (**d**). Nuclei are counterstained with 4′,6-diamidino-2-phenylindole (*DAPI*) (blue). Transmission electron microscopy images of human pericardium showing its ultrastructure after decellularization and lyophilization processes (**e** and **f**). Scale bars = 100 μm (a-c), 50 μm (d), 0.5 μm (e), and 200 nm (f).

## Abbreviations

CO: Cardiac output
ECM: Extracellular matrix
H/E: Hematoxylin/eosin
LVEF: Left ventricular ejection fraction
MI: Myocardial infarction
MRI: Magnetic resonance imaging
SMA: Smooth muscle actin
TEM: Transmission electron microscopy
vWF: Von Willebrand factor
