# Supplementary material for: Neoinnervation and neovascularization of acellular pericardial-derived scaffolds in myocardial infarcts
Source: Stem Cell Res Ther. 2015 May 27;6(1):108. doi: 10.1186/s13287-015-0101-6 (PMC4529715; doi:10.1186/s13287-015-0101-6)
Supplement: Additional file 1: — Supplemental Material. [file 13287_2015_101_MOESM1_ESM.docx]

**Supplemental Material**

**DETAILED METHODS**

This study was approved by the local Animal Experimentation Unit Ethical Committee and complies with all guidelines concerning the use of animals in research and teaching as defined by the Guide for the Care and Use of Laboratory Animals (NIH Publication No. 80–23, revised 1996). Human pericardial samples were obtained after written informed consent was obtained from all patients undergoing cardiac surgery. The local ethics committee approved this study, and the protocol conformed to the principles outlined in Declaration of Helsinki.

**Pericardial decellularization**

The pericardium was decellularized as previously described (Prat-Vidal *et al*., 2014). Briefly, adipose tissue from the pericardium was excised and, after cleaning with phosphate buffered saline (PBS), the tissues were washed for 72 h in detergent supplemented with 1% sodium dodecyl sulfate (Sigma). Then, the pericardia were rinsed with 1% Triton X-100 for 48 h at room temperature under stirring to facilitate cell removal and solutions were replaced every 24 h. Finally, decellularized human pericardia were cleaned 3 times within 24 h using PBS with 1% penicillin-streptomycin and treated with 0.1 mg/mL DNase (Roche) for 3 additional days. The resulting scaffolds were washed in PBS and lyophilized by drying under vacuum at −25°C for 24 h in a freeze-dry system (Christ loc-1m, B. Braun Biotech International), individually sealed, and sterilized using 25 kGy gamma irradiation at the standard dose for medical devices (ISO 11137-2:2012).

**Myocardial infarct induction**

Seventeen Landrace X Large White pigs (20-30 kg) were included and were premedicated and anesthetized as previously decribed (Gálvez-Montón *et al*., 2013). Briefly, propofol (4 mg/kg, IV; Recofol^®^, Bayer Schering Pharma) was used as an anesthetic and anesthesia was maintained by 2% sevoflurane inhalation. A continued IV infusion of tramadol (0.5 mg/kg/h; Adolonta^®^, Grünenthal Pharma, S.A.) and ketorolac (0.15 mg/kg/h; Toradol^®^, Roche) was used as surgical analgesia. At the beginning of the surgery, a 1 mg/kg lidocaine bolus (IV, Lidocaine^®^ 2%, B. Braun) was administered, followed by a continuous infusion (1 mg/kg/h) that was maintained for 1 h after myocardial infarct (MI) induction. Finally, as a post-operative antibiotic therapy, an intramuscular dose of 2.5 mg/kg tulathromycin (Draxxin^®^, Pfizer Animal Health, Madrid, Spain) was administered. After a lateral thoracotomy in the fourth intercostal space, all pigs were submitted to a MI by a double ligation of the first marginal branch of the circumflex coronary artery. Thirty minutes later, the cell-free, decellularized, human pericardium rehydrated with RAD16-I peptide was adhered over the ischemic area with cardiac surgical glue (Glubrand^®^2, Cardiolink) (Prat-Vidal *et al*., 2014). Finally, the surgical wound was reduced and the animals were housed for 30 days until sacrifice. All surgical procedures were done under monitoring conditions with capnography, electrocardiography, pulse oximetry, non-invasive arterial blood pressure, and temperature. The onset of ST segment elevation on the ECG and the damage to the affected myocardium were used to assess MI.

**Magnetic Resonance Imaging**

In a subgroup of pigs (n=7), the effect of the pericardial-derived scaffolds on cardiac function was assessed by magnetic resonance imaging (MRI). Cardiac function was monitored at baseline, 48 hours post-MI and after 1 month, before sacrifice. Breath-held, ECG-gated MRI images were acquired with a four-channel phase array, 1.5T MR Scanner (Intera, Philips, Best, The Netherlands). To assess LV global function, T1 short-axis images were taken using a standard steady-state free-precession sequence with a number of slices to cover the entire LV from apex to base. Imaging parameters were as follow: repetition time, 4.1 ms; echo delay time 2.1 ms; flip angle, 60°; field of view, 320×320 mm; matrix, 160×160 pixels, slice thickness, 7 mm; bandwidth, 1249.7 Hz/pixel). Delayed enhancement images were acquired 10 minutes after intravenous gadolinium bolus administration (Gd-DTPA, 0.2 mmol/kg; Magnevist®, Schering AG, Berlin, Germany) using a phase sensitive inversion-recovery sequence (repetition time, 4.9 ms; echo delay time 1.6 ms; flip angle, 15°; inversion time, 157 ms; field of view, 330×330 mm; matrix, 224×200 pixels; slice thickness, 10 mm, bandwidth, 282.3 Hz/pixel). Left ventricular ejection fraction (LVEF), cardiac output (CO), stroke volume (SV), end-diastolic volume (EDV), end-systolic volume (ESV), end diastolic wall mass (EDWM), and infarct size measurements were blindly analyzed on an offline workstation.

**Histological analysis and immunohistochemistry**

After sacrifice with an overdose of anesthesia, the hearts were excised to obtain samples including the MI area, and the attached cardiac engineered tissue for microscopic analysis. Samples were either fixed in 10% formalin and embedded in paraffin, or snap-frozen to obtain 4-μm or 10-μm slices, respectively. Histological analysis was done after hematoxylin/eosin (H/E) and Gallego’s modified and Masson’s trichrome staining in paraffin-embedded sections. Afterwards, snap-frozen sections were labeled with anti-β_III_ tubulin (1:500; Covance), anti-S100 (1:5000; Dako), anti-smooth muscle actin (SMA) (1:200; Abcam), anti-CD31 (1:50; Abcam), anti-Von Willebrand factor (vWF) (1:200; Abcam), anti-cardiac troponin I (cTnI) (1:100; Abcam), anti-Collagen I (1:100, Abcam), and anti-elastin (1:100; Abcam) antibodies and with biotinylated *Griffonia simplicifolia* lectin I (GSLI) B4 isolectin (1:50; Vector labs).

**Transmission electron microscopy**

Pericardial-derived scaffold samples were fixed in 2.5% glutaraldehyde in 0.1 M phosphate buffer, post fixed in 1.5% osmium tetroxide, and processed using the tEPON 812 embedding Kit (Tousimis^®^). The ultrathin sections, stained with uranyl acetate and lead citrate, were assessed at 80 kv with a JEM-1011 (JEOL) transmission electron microscope.

**REFERENCES**

1. Gálvez-Montón C, Prat-Vidal C, Roura S, Soler-Botija C, Llucià-Valldeperas A, Díaz-Güemes I, et al. Post-infarction scar coverage using a pericardial-derived vascular adipose flap. Pre-clinical results. Int J Cardiol. 2013;166:469-74.
2. Prat-Vidal C, Gálvez-Montón C, Puig-Sanvicens V, Sanchez B, Díaz-Güemes I, Bogonez-Franco P, et al. Online monitoring of myocardial bioprosthesis for cardiac repair. Int J Cardiol. 2014;174:654-61
